# Efficacy of Apatinib plus Temozolomide in Treating Recurrent Malignant Brain Glioma

**DOI:** 10.4314/ahs.v25i2.17

**Published:** 2025-06

**Authors:** Guoquan Li, Tao Hu, Yangwu Hu, Hailiang Shi, Ling Li, Gang Xiong

**Affiliations:** Department of Neurosurgery, Wuhan Jiangxia District First People's Hospital, Wuhan, China

**Keywords:** apatinib, temozolomide, recurrence, glioma, efficacy

## Abstract

**Background:**

To evaluate the efficacy and safety of apatinib plus tenozolomide in the treatment of recurrent malignant brain glioma.

**Methods:**

A retrospective analysis of 108 patients with recurrent malignant brain glioma was conducted at our hospital. Out of these, 54 patients received apatinib plus tenozolomide as combination therapy (Combination group) while the remaining 54 were treated with temozolomide alone (Control group). Clinical data was collected and analyzed to compare the treatment efficacy and incidence of adverse reactions between the two groups. Additionally, patient survival and progression-free survival (PFS) were monitored and recorded.

**Results:**

The study evaluated the efficacy of a treatment among patients who completed it. The Combination group had a higher objective response rate (ORR) and disease control rate (DCR) compared to the Control group. Treatment-related adverse reactions were mostly grade I-II and improved with symptomatic treatment. The Combination group had higher incidence rates of hypertension, proteinuria, and hand-foot syndrome. Follow-up results showed that the Combination group had a significantly better overall survival (OS) and progression-free survival (PFS) than the Control group.

**Conclusion:**

Compared with temozolomide alone, apatinib plus tenozolomide can significantly improve the clinical efficacy in treating recurrent malignant brain glioma, prolong the survival of patients and produce tolerable adverse reactions.

## Introduction

Brain glioma, a clinically common disease, is very difficult to treat or remove completely through surgery, and despite standard post-operative radiotherapy and chemotherapy, there have still been most patients suffering from recurrence, resulting in poor efficacy and no standard treatment strategy^1^,^2^. As an anti-tumor chemotherapeutic drug, temozolomide has been widely applied to intracranial tumors at present for its strong ability to penetrate the blood-brain barrier^3^. However, temozolomide is far from satisfactory in terms of overall response rate in treating recurrent glioma. Brain glioma is rich in vascular endothelial growth factor (VEGF), whose over-expresion is associated with high malignancy and poor prognosis^4^-^6^. Therefore, anti-VEGF antibodies, such as bevacizumab and small-molecule inhibitors against VEGF (like apatinib), have been used for treating patients with glioma^7^. Apatinib, a small-molecule targeted drug independently researched and developed in China, has produced preferable clinical efficacy in multiple malignant tumors, such as gastric cancer and lung cancer^8^,^9^. According to a study report, temozolomide plus apatinib has good clinical efficacy in treating recurrent gliomas^10^. In the present study, the clinical efficacy of temozolomide plus apatinib in treating recurrent malignant brain glioma and its influence on the immune function of patients as well as its safety were explored.

## Materials and methods

### General data

A total of 108 patients histologically diagnosed with recurrent malignant brain glioma and treated in our hospital were enrolled. All the patients received surgical treatment and post-operative standard Stupp protocol-based chemotherapy and concurrent radiotherapy, resulting in a mean post-operative recurrence time of 8.7 months. The inclusion criteria were set as follows: 1) patients undergoing surgical section and pathologically diagnosed with the World Health Organization grade III or IV brain glioma, 2) those who had measurable lesions as verified by magnetic resonance imaging (MRI), and 3) those with Karnowski performance scale score ≥60 points and life expectancy >3 months. The exclusion criteria involved the following patients: those complicated with severe dysfunctions of major organs, such as the heart, liver, kidney or lung, those who suffered from nervous-mental system diseases or could not cooperate in the treatment, those with concurrent blood diseases, endocrine system diseases, autoimmune diseases, or abnormal bone marrow reserve, or those complicated with other primary tumors. All the patients were divided into apatinib plus tenozolomide treatment group (Combination group, n=54) and simple temozolomide treatment group (Control group, n=54) based on different treatment regimens. Among them, 62 were males and 46 were females, with a mean age of (43.6±9.1) years old, and the pre-treatment baseline data showed no statistically significant differences between two groups of patients (P>0.05) (Table 1). The present study was approved by the Ethics Committee of our hospital and performed based on the Declaration of Helsinki, and all the patients were informed of the study and signed the informed consent.

**Table 1:** Baseline characteristics of the studied patients

Parameters	Combination group n=54	Control group n=54	*P*-value
Age (years)	44.03 ±8.48	42.81 ±9.19	0.475
Gender (Male/Female)	29/25	33/21	0.560
Tumor grade			0.562
III	22 (40.7%)	26 (48.1%)	
IV	32 (59.3%)	28 (51.9%)	
Number of tumor			0.391
Single	49 (90.7%)	45 (83.3%)	
Multiple	5 (9.3%)	9 (16.7%)	
Mean recurrence time (months)	8.9±4.5	8.4±4.8	0.578
KP S score (points)			0.418
≥70	38 (70.4%)	33 (61.1%)	
<70	16 (29.6%)	21 (38.9%)	

**Notes:** KPS: Karnofsky performance status.

### Treatment schemes

The patients in Control group were orally administrated with temozolomide at 150 mg/m2, once/d for 5 consecutive days, with 28 d as a cycle. In Combination group, apatinib was orally given at 250 mg once/d besides the temozolomide treatment for 12 consecutive cycles or until the patients had tumor progression or intolerable adverse reactions.

### Observation indicators

At 2 months after treatment, efficacy was evaluated by contrast-enhanced head MRI based on the response assessment in neuro-oncology criteria which includes complete remission (CR), partial remission (PR), stable disease (SD), and progressive disease (PD)^11^. The response rate = (CR+PR) cases/total cases ×100%, and disease control rate (DCR) = (CR+PR+SD) cases/total cases ×100%. Functional head MRI was conducted again every 2-3 months or in case of new symptoms, and blood, stool and urine routine test parameters, liver and kidney functions and coagulation function were reexamined every week during treatment. If proteinuria was tested positive, 24-huantification of proteinuria would be performed. After treatment, the changes in blood pressure of patients were monitored daily until drug discontinuation or death of patients. The follow-up ended in May 2021. The adverse reactions during treatment were recorded and assessed according to the Common Terminology Criteria Adverse Events Version 4.0 formulated by the National Cancer Institute (NCI). The survival of the patients was recorded. Progression-free survival (PFS) referred to the duration from the day of surgical treatment to the occurrence of PD or the latest follow-up, while the overall survival (OS) meant the duration from the day of surgical treatment to the death of patients or the latest follow-up.

### Statistical analysis

Statistical Product and Service Solutions (SPSS) 22.0 software (IBM, Armonk, NY, USA) was used for statistical analysis. Measurement data were presented as mean ± standard deviation (χ̅±s), and inter-group comparisons were made using two-sample t test. Besides, clinical data were compared with *χ*^2^ test or Fisher's exact test. The short-term efficacy and adverse reactions were compared as unidirectional ordered ranked data and examined using the Mann-Whitney U test. Patient's survival was analyzed using the Kaplan-Meier curve, and subjected to the log-rank test. P<0.05 suggested statistically significant differences.

## Results

### Comparison of short-term efficacy

The efficacy was evaluated in all the patients who completed the treatments. According to the results, Combination group had 13 cases of CR (24.1%), 22 cases of PR (40.7%), 12 cases of SD (22.2%) and 7 cases of PD (13.0%), with an objective response rate (ORR) of 64.8% (35/54) and a DCR of 87.0% (47/54), while Control group exhibited 8 cases of CR (14.8%), 15 cases of PR (27.8%), 14 cases of SD (25.9%) and 17 cases of PD (31.5%), with an ORR of 42.6% (23/54) and a DCR of 68.5% (37/54). The ORR and DCR in Combination group were substantially higher than those in Control group, showing statistically significant differences (P=0.033, P=0.036) (Table 2).

**Table 2:** Comparison of tumor response of patients in the two studied groups

Parameters	Combination group n=54	Control group n=54	*P*-value
Complete response (CR)	13 (24.1%)	8 (14.8%)	
Partial response (PR)	22 (40.7%)	15 (27.8%)	
Stable disease (SD)	12 (22.2%)	14 (25.9%)	
Progressive disease (PD)	7 (13.0%)	17 (31.5%)	
Objective response rate (ORR)	35 (64.8%)	23 (42.6%)	0.033
Disease control rate (DCR)	47 (87.0%)	37 (68.5%)	0.036

### Comparisons of adverse reactions

The patients in the two groups suffered from treatment-related adverse reactions, mainly manifested as bone marrow depression, nausea and vomiting, diarrhea, fatigue, hypertension, proteinuria and hand-foot syndrome, most of which were grade I-II, and improved after symptomatic treatment. Combination group had significantly higher incidence rates of hypertension, proteinuria and hand-foot syndrome than Control group (31.5% vs. 5.6%, P<0.001, 14.8% vs. 1.9%, P=0.031, 16.7% vs. 1.9%, P=0.016), which may be associated with the administration of apatinib, while there were no statistically significant differences in the other adverse reactions (P>0.05) (Table 3).

**Table 3:** Comparison of adverse reactions of patients in the two studied groups

Parameters	Combination group n=54	Control group n=54	*P*-value
Leukopenia	7 (13.0%)	5 (9.3%)	0.661
Anemia	5 (9.3%)	5 (9.3%)	1.000
Thrombocytopenia	6 (11.1%)	6 (11.1%)	1.000
Nausea and vomiting	14 (25.9%)	11 (20.4%)	0.549
Diarrhea	4 (7.4%)	3 (5.6%)	0.678
Fatigue	16 (29.6%)	13 (24.1%)	0.565
Hypertension	17 (31.5%)	3 (5.6%)	0.001
Proteinuria	8 (14.8%)	1 (1.9%)	0.031
Hand-foot syndrome	9 (16.7%)	1 (1.9%)	0.016

### Follow-up results of patients' survival

**Figure 1:**
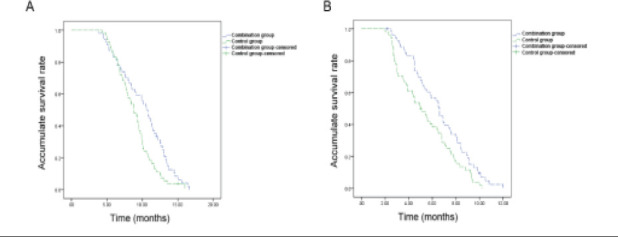
Kaplan-Meier survival curves of recurrent malignant glioma patients. The overall survival rate (A) and progression free survival rate (B) of patients in Combination group were significantly higher than those of Control group (P=0.011, P=0.017)

All the patients were followed up for 4-20 months until May 2021. According to the results, the median OS and PFS were (11.7±4.3) months and (7.4±2.7) months, respectively, in Combination group and (8.5±4.1) months and (4.9±2.5) months, respectively, in Control group. The survival curves of patients were plotted using the Kaplan-Meier method (Figure 1). The log-rank test results revealed that there were statistically significant differences in the OS and PFS between the two groups of patients, and the OS and PFS in Combination group were superior to those in Control group (P=0.011, P=0.017).

## Discussion

Glioma, a primary central nervous system malignancy, has very high recurrence and mortality rates. Despite routine postoperative radiotherapy and chemotherapy, its recurrence rate remains stubbornly high. Temozolomide, a new type of alkylating agent, can easily pass through the blood-brain barrier and be fully absorbed orally, serving as a broad-spectrum anti-tumor drug^3^. Oral administration of temozolomide has currently become a conventional treatment scheme for patients with postoperative recurrent gliomas, but it has poor efficacy, resulting in a median survival of about 1 year^12^. Therefore, seeking efficacious treatment regimens has become a research hotspot for recurrent brain glioma. VEGF and VEGFR that are excessively expressed on the surface of various tumor cells are important ligand and receptor affecting angiogenesis, respectively. Anti-angiogenic therapy can improve tumor microenvironment, thereby promoting normalization of tumor blood vessels. Such an effect may be the basis for the benefits of combined antiangiogenic and cytotoxic therapy^13^,^14^. Apatinib, a novel small-molecule tyrosine kinase inhibitor, mainly highly selectively and powerfully inhibits the phosphorylation of VEGF-2 and the activation of downstream ERK-1/2-MAPK and PDK-AKT-mTOR pathways to block signaling and repress tumor neovascularization, thereby inhibiting tumor growth. In addition, apatinib also suppresses c-kit, c-Src, platelet-derived growth factor receptor-β, and MET, thus directly exerting the anti-tumor effects^15^,^16^. It has been uncovered through studies in China and abroad that apatinib has significant anti-tumor activity in many malignant tumors, such as gastric cancer and lung cancer^17^. In recent years, apatinib plus chemotherapy has obtained some efficacy in patients with recurrent malignant brain glioma. Wang et al. discovered through a study that apatinib combined with temozolomide group had an ORR of 45% (9/20), a DCR of 90% (18/20), and a median OS of 9 months, which were obviously better than those in apatinib group or temozolomide group^18^. It has been proven that apatinib can enhance the sensibility of temozolomide, and they produce a synergistic anti-tumor effect together^19^.

The results of this study showed that the ORR and DCR were 64.8% (35/54) and 87.0% (47/54), respectively, in Combination group, which were considerably higher than those in Control group (P=0.033, P=0.036). Besides, the median OS and PFS were (11.7±4.3) months and (7.4±2.7) months, respectively, in Combination group and (8.5±4.1) months and (4.9±2.5) months, respectively, in Control group. According to the follow-up results, Combination group was remarkably superior to Control group in OS and PFS (P=0.011, P=0.017). In terms of safety, as an antiangiogenic agent, apatinib often produces such adverse reactions as hypertension, proteinuria, hand-foot syndrome, fatigue and bone marrow inhibition. According to a report, the incidence rates of grade III hypertension, hand-foot syndrome and proteinuria are 5.38%, 7.62%, and 1.79%, respectively, while the incidence rates of neutropenia, thrombocytopenia, leucopenia, gastrointestinal reaction and fatigue, which are the common grade III-IV adverse reactions of temozolomide, are 5.9%, 9.7%, 5.7%, 1.6%, and 5.7%, respectively^20^. There were grade III or above adverse reactions in the present study, and all the adverse reactions were relieved through symptomatic treatment, indicating good tolerance and safety of combined medication. Combination group had markedly higher incidence rates of hypertension, proteinuria, and hand-foot syndrome than control group, which might be correlated with the administation of apatinib. However, this study also has certain limitations. Firstly, the sample size was small, which may limit the generalizability of the study results and reduce the statistical power. Secondly, the follow-up time was short, which might restrict the ability to observe long-term outcomes and assess the durability of the intervention or treatment being studied. Lastly, the follow-up content was not comprehensive enough. Therefore, in the future, we will overcome these limitations by conducting multi-center, large-sample, prospective randomized controlled trials to provide reliable evidence for drug treatment of recurrent malignant gliomas.

## Conclusion

Compared with temozolomide alone, apatinib plus tenozolomide can significantly improve the clinical efficacy in treating recurrent malignant brain glioma, prolong the survival of patients and produce tolerable adverse reactions.
